# Schizophrenia Biomarkers: Blood Transcriptome Suggests Two Molecular Subtypes

**DOI:** 10.1007/s12017-024-08817-x

**Published:** 2024-11-28

**Authors:** Herut Dor, Libi Hertzberg

**Affiliations:** 1 The Faculty of Medical and Health Sciences, Tel Aviv University, Tel Aviv, Israel; 2 Department of Physics of Complex Systems, Weizmann Institute of Science, 76100 Rehovot, Israel; 3 Shalvata Mental Health Center, Affiliated with the Faculty of Medicine, Tel-Aviv University, 13 Aliat Hanoar St., 45100 Hod Hasharon, Israel

**Keywords:** Gene expression, Unsupervised learning, Pathway enrichment analysis, Ribosome, Ubiquitin proteasome system

## Abstract

Schizophrenia is a chronic illness that imposes a significant burden on patients, their families, and the health care system. While it has a substantial genetic component, its heterogeneous nature—both genetic and clinical—limits the ability to identify causal genes and mechanisms. In this study, we analyzed the blood transcriptomes of 398 samples (212 patients with schizophrenia and 186 controls) obtained from five public datasets. We demonstrated this heterogeneity by clustering patients with schizophrenia into two molecular subtypes using an unsupervised machine-learning algorithm. We found that the genes most influential in clustering were enriched in pathways related to the ribosome and ubiquitin-proteasomes system, which are known to be associated with schizophrenia. Based on the expression levels of these genes, we developed a logistic regression model capable of predicting schizophrenia samples in unrelated datasets with a positive predictive value of 64% (*p* value = 0.039). In the future, integrating blood transcriptomics with clinical characteristics may enable the definition of distinct molecular subtypes, leading to a better understanding of schizophrenia pathophysiology and aiding in the development of personalized drugs and treatment options.

**Supplementary Information:**

The online version contains supplementary material available at 10.1007/s12017-024-08817-x.

## Introduction

For decades, psychiatric disorders—and schizophrenia in particular—have remained unresolved in terms of their etiology, pathophysiology, and effective treatment.

Schizophrenia is a brain disease defined by a combination of positive symptoms (e.g., hallucinations) and negative symptoms (e.g., flat affect). It is known that schizophrenia has a substantial hereditary component, estimated at 80% (Hilker et al., 2018), which is higher than most non-psychiatric diseases.

Genome-Wide Association Studies (GWAS) have identified more than 280 genetic variants associated with schizophrenia, each contributing a small increase in risk (Trubetskoy et al., 2022; Zeng et al., 2021). Many of these variants are located within regulatory sequences that affect gene expression rather than protein structure (Roussos et al., 2014), highlighting the importance of examining gene expression patterns rather than just the genetic sequence.

A possible explanation for the difficulty in understanding the genetics of schizophrenia and the mechanisms underlying its development is that schizophrenia may be a group of several distinct diseases, each with unique genetics, pathophysiology, gene expression patterns, and response to treatment (Ahmed et al., 2018; Hertzberg et al., 2021; Tsuang et al., 2001). This point of view had even appeared in previous versions of the Diagnostic and Statistical Manual of Mental Disorders (DSM) definition of schizophrenia but was later abandoned due to a lack of evidence (Mattila et al., 2015).

Recently, the notion of schizophrenia subtypes has been suggested based on gene expression analyses of brain tissue. Several subtypes have been described in the literature based on genes related to the ribosome (Mekiten et al., 2023), the Ubiquitin Proteasome System (UPS) (Bowen et al., 2019; Hertzberg et al., 2021), and immune-related cytokines (Fillman et al., 2016; Goldsmith et al., 2016), although the relationships between these subtypes have not yet been examined. To our knowledge, similar results have not yet been observed in blood samples. While elevated inflammatory markers have been identified in a subgroup of patients in both the brain and blood, the interplay between peripheral inflammation and neuroinflammation remains unclear, and the limited strength of the identified potential blood biomarkers does not allow clinical use (Bishop et al., 2022). Studies of gene expression in brain samples have inherent limitations. These samples may have been altered by postmortem processes, may not be indicative of the young adults in whom schizophrenia begins, and cannot serve as clinical biomarkers. This emphasizes the need to search for blood biomarkers that can be monitored throughout a patient's life, reflecting both baseline expression levels and changes over time. Such biomarkers may enable the subtyping of patients with schizophrenia, which is highly relevant to both clinical and basic research studies and may serve as diagnostic and clinical tools.

In this paper, we examined the heterogeneity hypothesis using gene expression data obtained from blood samples of individuals with schizophrenia. We found that patients with schizophrenia can be clustered into two molecular subtypes. One subtype had gene expression levels similar to those of the healthy controls, while the other subtype showed gene expression levels that differed substantially from those of the healthy controls. This finding supports the hypothesis that heterogeneity is a main cause of the difficulty in identifying causal genes and mechanisms involved in the development of schizophrenia. Next, we sought to establish the roles of the genes whose expression varied the most between the two subtypes. These genes include those related to the ribosome and the Ubiquitin Proteasome System (UPS), both known to be associated with schizophrenia (Mekiten et al., 2023; Veiko et al., 2003). To validate our findings, we predicted schizophrenia in blood samples obtained from individuals with schizophrenia and healthy controls based on ribosome and UPS genes in four unrelated datasets. In total, we analyzed 398 samples (212 patients with schizophrenia and 186 controls).

Further research is required to determine the relationship between the clinical variability of schizophrenia and the different molecular subtypes.

## Materials and Methods

The study utilized the public dataset GSE38484, obtained from the Gene Expression Omnibus (GEO) repository (https://www.ncbi.nlm.nih.gov/geo/query/acc.cgi?acc=GSE38484) (de Jong et al., 2012; Van Eijk et al., 2015), henceforth, referred to as the de Jong 2012 dataset. The dataset contains RNA sequencing data for 202 blood samples (106 patients with schizophrenia and 96 controls). For each sample, the available data include the expression levels for 48,743 probes, as well as age and sex. This dataset was preprocessed by the researchers who created it, including background correction in Beadstudio (v3.2.3), transformation (vst), and normalization (rsn) in Lumi R package (de Jong et al., 2012).

Validation of the model was carried out using blood gene expression from all available datasets in the GEO repository, comprising an additional 196 samples (106 patients with schizophrenia and 90 controls), taken from four datasets: GSE27383 (van Beveren et al., 2012), GSE38481 (de Jong et al., 2012), GSE18312 (Bousman et al., 2010b), and GSE48072 (Stoll et al., 2013). The details of each dataset are listed in Table 1.

**Table 1:** Characteristics of individual datasets used in the research

	Accession	Publication	# Schizophrenia samples	# Control samples	Platform	Mean age (standard deviation)
1	GSE38484, named in this paper de Jong 2012	de Jong et al. (2012)	106	96	Illumina HumanHT-12 V3.0 expression beadchip	39.5 (12.5)
2	GSE27383	van Beveren et al. (2012)	43	29	Affymetrix Human Genome U133 Plus 2.0 Array	No age information
3	GSE38481	de Jong et al. (2012)	15	22	Illumina HumanRef-8 v3.0 expression beadchip	30 (11)
4	GSE18312	Bousman et al. (2010b)	13	8	Affymetrix Human Exon 1.0 ST Array	44 (7.8)
5	GSE48072	Stoll et al. (2013)	35	31	Illumina HumanHT-12 V4.0 expression beadchip	No age information

### Pre-Processing

To obtain an expression matrix for each gene in each sample, we preprocessed the extracted gene expression data. Preprocessing included uniting duplicate probes for the same gene and removing genes with low variability, as these genes are expected to be expressed similarly across all samples, making them less informative. (Details about these processes can be found in the Supplementary Information).

### Clustering

Clustering of the schizophrenia samples of the de Jong 2012 dataset was performed with a subset of 2321 genes that were differentially expressed between the schizophrenia samples and the healthy controls (absolute value of log-fold change (FC) > 0.1, *p* value with FDR adjustment < 0.05). Among this subset of genes, 1125 genes were downregulated, and 1196 genes were upregulated in the patients with schizophrenia.

Clustering of the schizophrenia samples was performed using an unsupervised algorithm, specifically *k*-means with *k* = 2 (chosen using the elbow method Cui, 2020).

### Pathway Enrichment Analysis

To determine which genes had the greatest influence on clustering, we used the Silhouette coefficient, which estimates the quality of clustering based on the distance between clusters (Rousseeuw, 1987). The calculations were repeated each time without one of the genes. Genes with high significance in determining clustering had the greatest impact on this coefficient. Genes with Silhouette coefficients in the top third are regarded as the most important. Pathway enrichment analysis of these genes was conducted using the DAVID (The Database for Annotation, Visualization, and Integrated Discovery) tool (https://david.ncifcrf.gov/tools.jsp) (Huang et al., 2009a, 2009b; Sherman et al., 2022).

### Prediction of Which Samples Belong to a Patient with Schizophrenia in Four Unrelated Datasets

The additional four datasets were processed in the same manner as that described in the Methods section “Pre-Processing.”

For prediction, a logistic regression model was used (Hosmer et al., 2013). The model features were chosen based on gene groups enriched in the pathway enrichment analysis (namely ribosomes and UPS) which were present in both the training and testing datasets. The de Jong 2012 dataset was used to train this model, and four additional independent datasets were used to test it.

Because the number of features (189 genes) is in the order of the number of samples available for training the model (202 samples), we used Least Absolute Shrinkage and Selection Operator (LASSO) regression, a method of feature selection based on penalizing the absolute size of regression coefficients (Ranstam & Cook, 2018).

## Results

### Clustering of Schizophrenia Samples

**Fig. 1:**
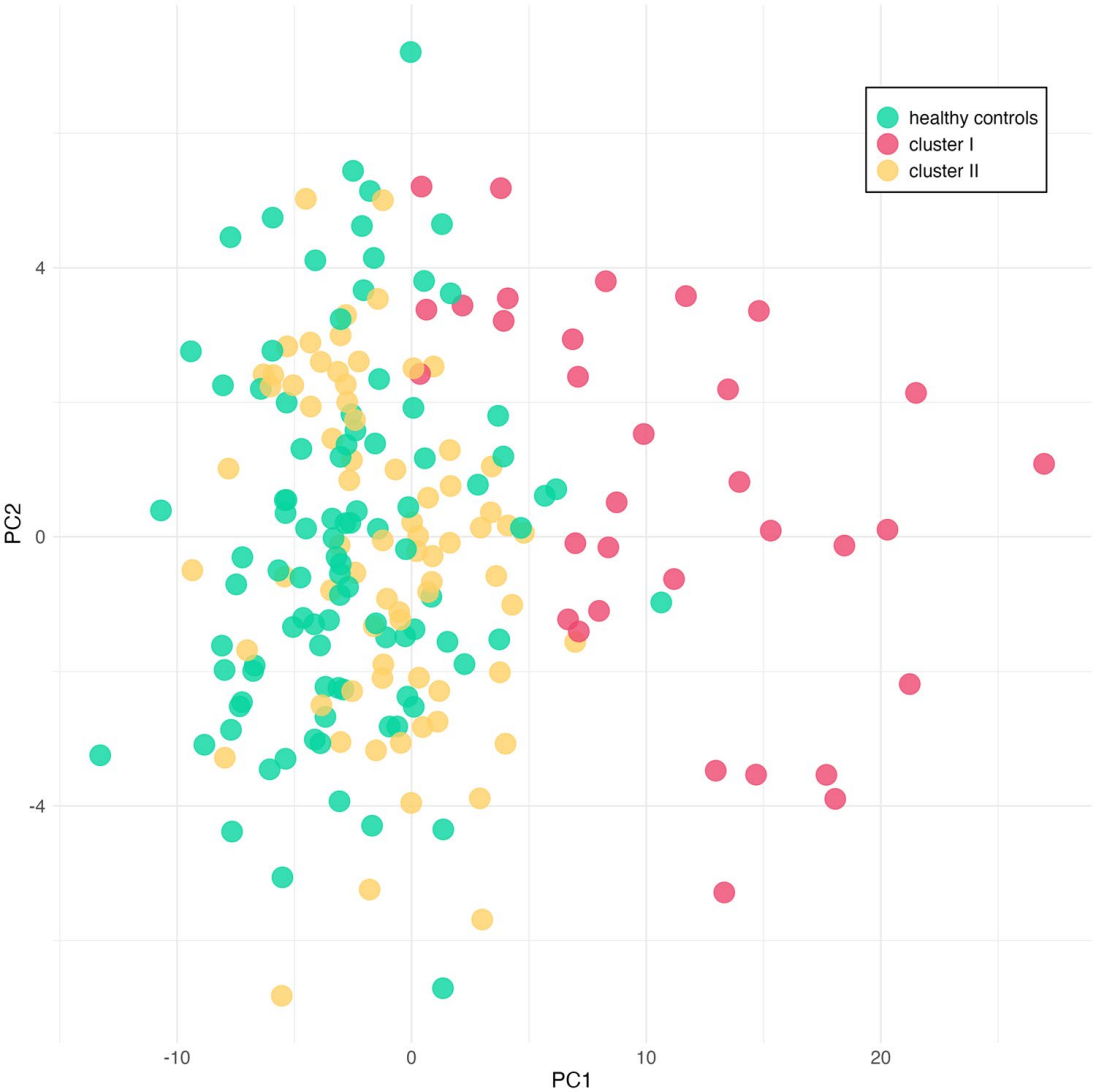
Principal Component Analysis (PCA) classification of the samples based on the ribosome genes level of expression. Each sample is color-coded according to its assigned group: Cluster I, Cluster II, or healthy controls. Cluster I predominantly consists of a distinct subset of schizophrenia samples

The k-means algorithm was applied to the 106 patients with schizophrenia from the de Jong 2012 dataset, using a subset of 2321 genes that were differentially expressed between the schizophrenia and control samples, revealing two clusters with distinct gene expression patterns: one with 33 samples (“Cluster I”) and the other with 73 samples (“Cluster II”) (Fig. 1). The clustering based on ribosomal genes is presented in Fig. 1. Similar results based on UPS genes are shown in Supplementary Figure 1. These groups differed in their expression levels relative to healthy controls.

Among the 2321 differentially expressed genes used for clustering, 97% were statistically significant (FDR adjusted *p* value < 0.05) in Cluster I (1155 were upregulated; 1099 were downregulated), compared with 43% in Cluster II (394 upregulated; 592 downregulated).

### Pathway Enrichment Analysis

To better understand the differential expression between the two clusters, we examined the 766 genes that were most important in determining the clustering (see Methods section “Pathway Enrichment Analysis”). Pathway enrichment analysis identified genes related to ribosomes, UPS, mitochondria, and messenger RNA (mRNA) processing (Supplementary Table 1). We focused on the ribosomal and UPS gene groups since only in these groups were all the related pathways statistically significantly enriched (Bonferroni adjusted *p* value < 0.05).

**Fig. 2:**
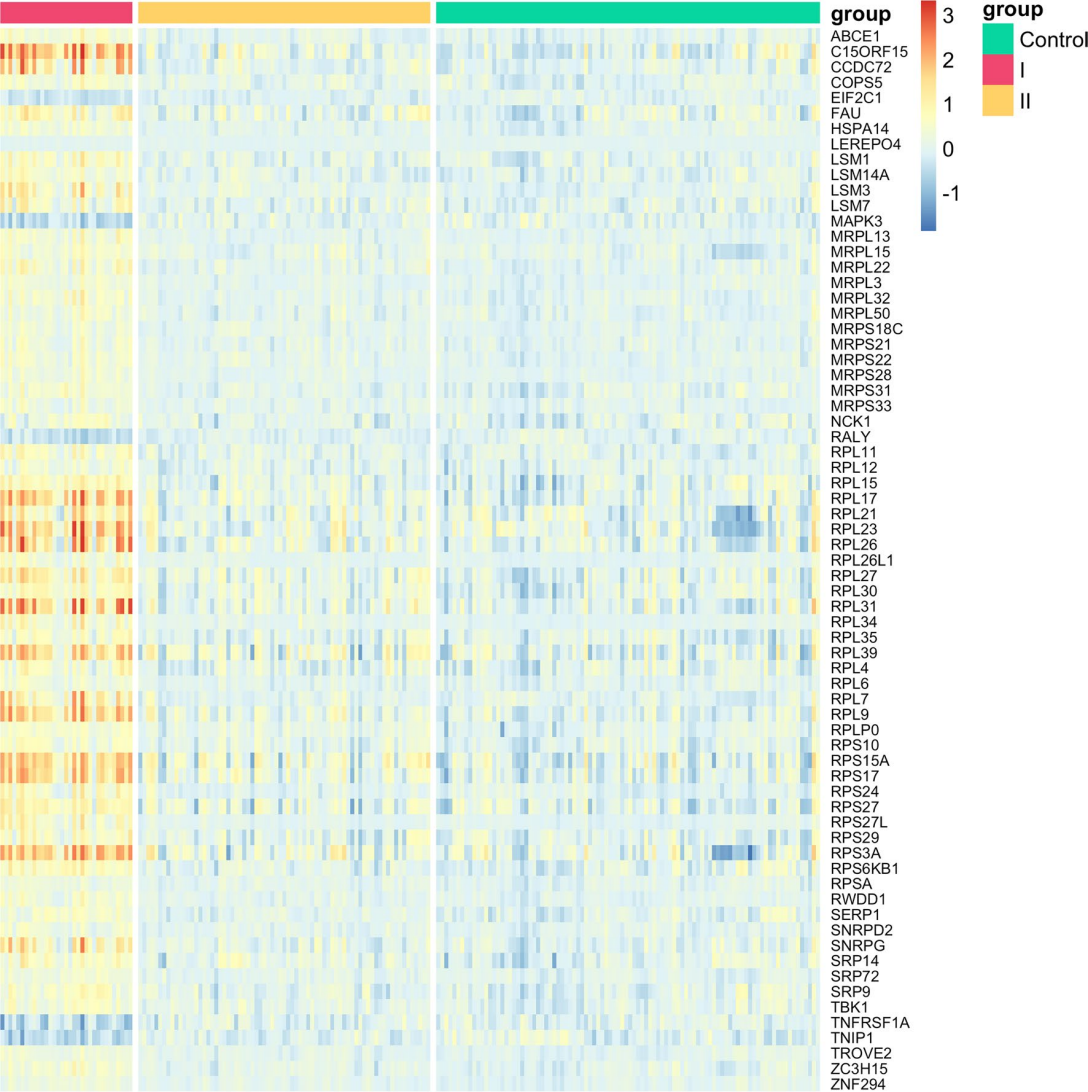
A heatmap shows the expression level of the ribosome genes in the patients with schizophrenia, relative to the healthy controls. The rows represent genes and the columns represent samples of patients with schizophrenia, color coded according to its assigned cluster. In the color map, the cell color is denoted in accordance with the level of expression as compared to the mean expression in the healthy control group ${C}_{\text{gene},\text{ sample}}=\frac{{\text{Expression}}_{\text{gene},\text{sample}}}{{<{\text{Expression}}_{\text{gene}}>}_{\text{controls}}}.$

Most of the enriched genes were found to be upregulated in patients with schizophrenia compared with healthy controls (Table 2). Notably, the majority of the differential expression of the ribosomal genes and almost all the UPS genes are explained by the difference between cluster I and healthy controls (99.5% of the genes are significantly differentially expressed between cluster I and controls, compared to 10% between cluster II and control). A heatmap showing the expression levels of ribosomal genes in patients with schizophrenia relative to healthy controls is presented in Fig. 2. Similar results based on UPS genes are shown in Supplementary Figure 2.

**Table 2:** Differences in gene expression between healthy controls and each cluster

	Schizophrenia vs. control	Cluster I vs. control	Cluster II vs. control
Ribosomal genes (69 genes)	68 (↑ 63, ↓ 5)	68 (↑ 63, ↓ 5)	16 (↑ 15, ↓ 1)
UPS genes (144 genes)	144 (↑ 117, ↓ 27)	144 (↑ 117, ↓ 27)	4 (↑ 2, ↓ 2)

Each cell contains the number of significantly differentially expressed genes (↑upregulated genes, ↓ downregulated genes)

### Dealing with Potential Confounding Factors

Potential confounding factors for the differences in expression between the two groups included age, sex, and use of antipsychotic therapy. We had age and sex information available for each dataset, allowing us to identify statistically significant differences by applying the t-test to age distribution and Fisher's exact test to the sex distribution. A summary of the main characteristics of the patients with schizophrenia in each cluster is presented in Table 3.

**Table 3:** Characteristics of the schizophrenia samples in each cluster of the de Jong 2012 dataset

	Cluster I (*N* = 33)	Cluster II (*N* = 73)	*p*-Value
Age—yr	42.8 ± 9.8	38.1 ± 10.9	0.03
Male sex—no. (%)	26 (0.79)	50 (0.68)	0.35

There was no significant difference in the sex ratio between the two clusters (*p* value = 0.35).

There was a significant difference in the age variable (*p* value = 0.03), but this difference was not expected to have clinical significance (43 ± 10 years vs. 38 ± 11 years). To examine the impact of this difference on clustering, we used a linear regression model to predict ribosomal and UPS gene expression levels based on the age and cluster of each sample. In this analysis, the age coefficients were all close to zero, while the cluster coefficients were much greater (Supplementary Fig. 3), suggesting that the differential expression between the two groups does not stem from a difference in the mean age.

### Genes Enriched in the Clustering Determination: Ribosomal Genes

Ribosomes are cell organelles that consist of proteins and ribosomal RNA (rRNA). They are responsible for translating mRNA sequences into polypeptides that eventually become proteins. Proteins are essential for all aspects of cellular function. Since all cellular proteins are synthesized by ribosomes, these organelles play a critical role in cell function. Brain diseases have been associated with impaired mRNA translation (Storkebaum et al., 2023). The expression patterns of genes encoding ribosomal proteins in the brain were found to be different between patients with schizophrenia and healthy individuals with a tendency toward upregulation (Mekiten et al., 2023). In addition, elevated rRNA transcription has been observed in the brain and lymphocytes of patients with schizophrenia (Veiko et al., 2003). Several explanations have been proposed for the association between schizophrenia and ribosomal genes. In patients with schizophrenia, increased oxidative stress might be associated with upregulated expression of ribosomal genes, to increase the production of proteins such as anti-oxidative enzymes that can help mitigate that stress (Porokhovnik et al., 2013). Another explanation is that malfunctioning ribosomes may lead to the accumulation of damaged proteins in the neurons of individuals with schizophrenia, a phenomenon observed in the postmortem brains of these individuals (Nucifora et al., 2019).

### Genes Enriched in the Clustering Determination: UPS Genes

The UPS is responsible for the degradation of proteins within the cell. Genetic variance (Chang et al., 2018; Pescosolido et al., 2013), altered transcription, (Altar et al., 2005; Arion et al., 2015; Bousman et al., 2010a; Hertzberg et al., 2021; Middleton et al., 2002), and altered protein levels (Rubio et al., 2013; Scott et al., 2015) of this system have been demonstrated to be associated with schizophrenia. The mechanism behind this connection is still unclear, but some studies have suggested that the accumulation of ubiquitinated proteins that do not undergo proper degradation contributes to the development of schizophrenia (Nucifora et al., 2019).

### Predict Samples of Schizophrenia in Unrelated Datasets

**Fig. 3:**
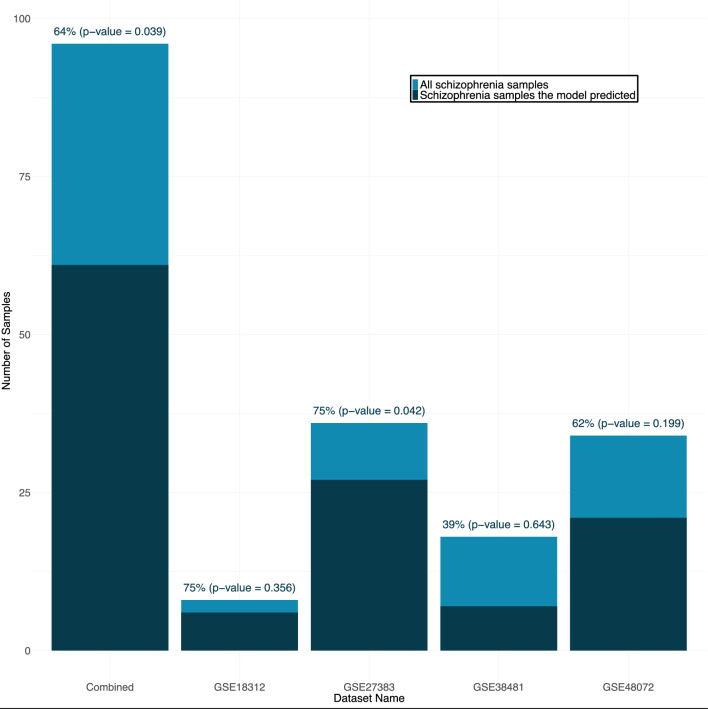
Bar plot of the Positive Predictive Value (PPV) for predicting schizophrenia samples in four unrelated datasets. Each bar represents a single dataset with the PPV displayed at the top of each bar

A logistic regression model was applied to four additional independent datasets (for details, please refer to Methods section “Prediction of Which Samples Belong to a Patient with Schizophrenia in Four Unrelated Datasets”). The model was evaluated using its positive predictive value (PPV), calculated using the number of true positives and false positives (TP, FP): TP/(TP + FP). We decided to use PPV as an indicator because a unique gene expression pattern characterizes a subgroup of patients with schizophrenia (“cluster I”) but not all of them. As this cluster exhibits a unique gene expression pattern, the model can predict schizophrenia samples with this distinctive expression. The PPVs for each of the four additional datasets were 75% (GSE27383), 39% (GSE38481), 75% (GSE18312), and 62% (GSE48072), as shown in Fig. 3. The combined PPV for all datasets was 64% (*p* value = 0.039). PPV may vary greatly across datasets and may not always be significant due to the relatively small number of schizophrenia samples in each dataset.

## Discussion

In this paper, we demonstrated the existence of two molecular subtypes based on blood transcriptome analysis of patients with schizophrenia: one with significant differential expression patterns compared to the healthy population and the other with gene expression patterns similar to those of healthy controls. We then found an enrichment of ribosomal and UPS genes in the list of genes that had the greatest influence on determining the molecular subtypes. The findings were replicated in four unrelated datasets, where these gene groups were used to identify one of the subtypes of patients with schizophrenia from a mixed group of healthy controls and individuals with schizophrenia.

The enriched genes were consistent with findings from earlier studies on brain tissue. Similar to our findings, (Bowen et al., 2019) identified two molecularly distinct types using dorsolateral prefrontal cortex (DLPFC) transcriptome data. Among the two types, one has a transcriptome that closely resembles that of the controls, whereas the other has a transcriptome that is strikingly different. This study also identified enrichment of the UPS and mitochondrial pathways among the genes most important for clustering. UPS gene enrichment among differentially expressed genes was also found in a study by (Hertzberg et al., 2021), and ribosomal gene upregulation was found in a meta-analysis by Mekiten et al. (2023) in various brain regions.

In light of all this evidence from different tissues, the ribosome and UPS genes may play a role both as potential biomarkers and possibly in the development of schizophrenia.

Our study's results must be viewed within the context of the following limitations. First, since the datasets are cross-sectional, they cannot demonstrate causality, only association. Ongoing research on the pathophysiology of the identified genes or blood samples collected at different stages of the disease could provide evidence of causality. Thus, our results are useful only as biomarkers. Second, the expression of genes in white blood cells can be influenced by various factors, including infection (Hu et al., 2013) and physical activity (Büttner et al., 2007), which may dilute the effects of schizophrenia. Treatment with antipsychotic drugs may also affect gene expression in the blood, as has been shown in the brain (Hoffman et al., 2019). Because of our limited understanding of all external factors affecting gene expression and the limited information available on the samples (only age and sex), it was not possible to exclude the effect of these potential confounding factors. Third, the direction of change in the UPS genes differed in the molecular subtypes identified using brain and blood samples. Although the genes identified as enriched were supported by earlier studies on brain tissue (Bowen et al., 2019; Hertzberg et al., 2021), these studies did not replicate the direction of change (i.e., upregulation or downregulation). UPS genes were predominantly upregulated in the blood samples examined, whereas they were mostly downregulated in the superior temporal gyrus region (Hertzberg et al., 2021). This discrepancy highlights the necessity of combining gene expression data from brain tissues to better understand the pathophysiology of schizophrenia.

There are still some open questions regarding the mechanisms by which ribosomal and UPS genes affect schizophrenia development. Some studies suggest that proteasome dysfunction in the UPS is involved (Scott & Meador-Woodruff, 2019), but other studies are inconsistent with this conclusion. It is also unclear whether the molecular heterogeneity found in this paper is consistent with the heterogeneity of clinical disease expression.

Combining blood transcriptomic profiling with clinical data (e.g., response to treatment) may enable us to connect different molecular subtypes to the clinical features of schizophrenia in the future. Oncology experienced a similar revolution about a decade ago, where instead of treating cancer in a single organ (e.g., lung cancer) as a single disease, treatment, and prognosis are now based on the genomic profile of the cancer cells (e.g., cancers with KRAS mutations and low PD-L1 expression). Using biomarkers for subtyping patients with schizophrenia may facilitate the development of personalized treatments. As in oncology, this direction has the potential to advance our understanding of the underlying biology and pathophysiology of the disease, and may be key to the development of objective diagnostic tools and more effective treatments. Further research on this topic is eagerly awaited.

## Conclusion

In summary, using publicly available gene expression datasets, we identified two groups of genes, ribosomes and UPS, which can be used to identify molecular subtypes of patients with schizophrenia. These results await replication by future research to determine whether a connection can be established between molecular subtypes and clinical schizophrenia characteristics. In addition, the results may serve as biomarkers for subtyping patients, potentially leading to improved diagnosis and treatment development.

## Supplementary Information

Below is the link to the electronic supplementary material.

Supplementary file1 (DOCX 937 KB)

## Data Availability

The data used to support the findings are available in the GEO dataset. The code used to create the results is available on GitHub at https://github.com/HerutDor/schizophrenia_biomarkers.git.

## Data Availability

The data are publicly available at the Gene Expression Omnibus (GEO) repository website (https://www.ncbi.nlm.nih.gov/geo/).
