# Supplementary material for: Schizophrenia Biomarkers: Blood Transcriptome Suggests Two Molecular Subtypes
Source: Neuromolecular Med. 2024 Nov 28;26(1):50. doi: 10.1007/s12017-024-08817-x (PMC11604812; doi:10.1007/s12017-024-08817-x)
Supplement: Supplementary file 1 — Supplementary file1 (DOCX 937 KB) [file 12017_2024_8817_MOESM1_ESM.docx]

**Supplementary Information for the papar “Schizophrenia biomarkers: blood transcriptome suggests two molecular subtypes”**

**NeuroMolecular Medicine**

Herut Dor^[[1]](#footnote-2)^, Libi Hertzberg^[[2]](#footnote-3),^^[[3]](#footnote-4)^

**Pre-Processing of the data**

To obtain an expression matrix for each gene in each sample, we preprocessed the extracted gene expression data.

1. Match probes to genes. This analysis was conducted using the annotation data included in the dataset.
2. Remove duplicate genes. In the original data, several probe-sets corresponded to the same gene. To obtain a unique expression for each gene, we averaged the expression across all probe-sets corresponding to the same gene. This method was chosen over replacing all the probe-sets with the one showing maximal expression since, after the VST transformation, the variance does not decrease as the level of expression increases. In the de Jong 2012 dataset, the expression data includes 37,776 genes for each sample.

Filter out genes with low variability. Low variability genes are expected to be expressed similarly in all the samples, making them less informative. Thus, we decided to exclude them from the analysis and focus on genes whose expression is variable. The cutoff standard deviation was set at 0.05. The results were not sensitive to a particular value of standard deviation. Using this filter on the dataset of de Jong 2012, 4,426 genes were removed, representing 12% of the total number of genes.

**Supplementary Fig 1.** Principal Component Analysis (PCA) classification of the samples based on the UPS genes level of expression. Each sample is color-coded according to its assigned group: Cluster I, Cluster II, or healthy controls. Cluster I predominantly consists of a distinct subset of schizophrenia samples.

**Supplementary Fig 2.** A heatmap shows the expression level of the UPS genes in the patients with schizophrenia, relative to the healthy controls. The rows represent genes, and the columns represent samples of patients with schizophrenia, color-coded according to its assigned cluster. In the color map, the cell color is denoted in accordance with the level of expression as compared to the mean expression in the healthy control group $C_{gene, sample}=\frac{Expression_{gene,sample}}{{<Expression_{gene}>}_{controls}}$.

**Supplementary Fig 3.** The magnitude of coefficient parameters used to predict the level of ribosome and UPS genes in the sample from patients with schizophrenia. A normalized age (age/10) and cluster of the sample are the model parameters. This graph indicates that all age coefficients are less than 0.1, while only 1.5% of cluster coefficients are less than 0.1.(S1a) The ribosomal genes, (S1b) the UPS genes.

**Supplementary Table 1.** The results of functional annotation clustering for genes contributing to subtyping determination. Functional annotation clustering was performed using the DAVID software. Each annotation cluster represents a specific gene group. In this table, we have presented the ten annotation clusters with the highest statistical significance. As shown in the table, the ribosome and UPS gene groups are the only annotation clusters in which all related pathways are enriched with a corrected p-value less than 0.05.

| **Functional Annotation Clustering** | | | | | | | |
| --- | --- | --- | --- | --- | --- | --- | --- |
| **Annotation Cluster 1** | **Enrichment Score: 15.4** |  |  |  |  |  |  |
| **Category** | **Term** | **Cnt.** | **%** | **P-Value** | **Bonferroni** | **Benjamini** | **FDR** |
| UP_KW_MOLECULAR_FUNCTION | Ribonucleoprotein | 51 | 7.2 | 9.47E-21 | 6.44E-19 | 6.44E-19 | 6.34E-19 |
| GOTERM_BP_DIRECT | cytoplasmic translation | 29 | 4.1 | 2.84E-20 | 7.18E-17 | 7.18E-17 | 7.17E-17 |
| UP_KW_MOLECULAR_FUNCTION | Ribosomal protein | 40 | 5.6 | 7.14E-20 | 4.85E-18 | 2.43E-18 | 2.39E-18 |
| GOTERM_MF_DIRECT | structural constituent of ribosome | 39 | 5.5 | 5.41E-19 | 3.90E-16 | 1.30E-16 | 1.30E-16 |
| GOTERM_CC_DIRECT | cytosolic ribosome | 27 | 3.8 | 8.49E-18 | 4.66E-15 | 2.33E-15 | 2.23E-15 |
| GOTERM_BP_DIRECT | translation | 39 | 5.5 | 1.06E-17 | 2.67E-14 | 1.34E-14 | 1.34E-14 |
| KEGG_PATHWAY | Ribosome | 36 | 5.1 | 1.37E-17 | 3.91E-15 | 3.91E-15 | 3.83E-15 |
| GOTERM_CC_DIRECT | ribosome | 33 | 4.7 | 9.19E-16 | 4.88E-13 | 1.68E-13 | 1.61E-13 |
| GOTERM_CC_DIRECT | cytosolic large ribosomal subunit | 19 | 2.7 | 2.69E-13 | 1.47E-10 | 2.95E-11 | 2.81E-11 |
| UP_KW_DISEASE | Diamond-Blackfan anemia | 11 | 1.6 | 1.18E-10 | 5.78E-09 | 5.78E-09 | 5.78E-09 |
| GOTERM_CC_DIRECT | ribonucleoprotein complex | 34 | 4.8 | 1.63E-10 | 8.97E-08 | 1.49E-08 | 1.43E-08 |
| KEGG_PATHWAY | Coronavirus disease - COVID-19 | 32 | 4.5 | 2.18E-10 | 6.24E-08 | 3.12E-08 | 3.06E-08 |
|  |  |  |  |  |  |  |  |
| **Annotation Cluster 2** | **Enrichment Score: 4.5** |  |  |  |  |  |  |
| **Category** | **Term** | **Cnt.** | **%** | **P-Value** | **Bonferroni** | **Benjamini** | **FDR** |
| UP_SEQ_FEATURE | CROSSLNK:Glycyl lysine isopeptide (Lys-Gly) (interchain with G-Cter in SUMO2) | 76 | 10.7 | 1.97E-07 | 5.21E-04 | 5.21E-04 | 5.21E-04 |
| UP_KW_PTM | Isopeptide bond | 102 | 14.4 | 2.08E-04 | 0.00496995 | 0.0017298 | 0.0017298 |
| UP_KW_PTM | Ubl conjugation | 135 | 19 | 5.66E-04 | 0.01348714 | 0.00353518 | 0.00353518 |
|  |  |  |  |  |  |  |  |
| **Annotation Cluster 3** | **Enrichment Score: 3.1** |  |  |  |  |  |  |
| **Category** | **Term** | **Cnt.** | **%** | **P-Value** | **Bonferroni** | **Benjamini** | **FDR** |
| UP_KW_CELLULAR_COMPONENT | Mitochondrion | 76 | 10.7 | 2.63E-05 | 0.00105309 | 3.51E-04 | 3.16E-04 |
| GOTERM_CC_DIRECT | mitochondrial inner membrane | 35 | 4.9 | 2.83E-05 | 0.01541887 | 0.00119529 | 0.00114086 |
| UP_KW_DOMAIN | Transit peptide | 32 | 4.5 | 4.32E-04 | 0.01075539 | 0.01081131 | 0.01081131 |
| UP_SEQ_FEATURE | TRANSIT:Mitochondrion | 32 | 4.5 | 0.0020358 | 0.99541093 | 1 | 1 |
| GOTERM_CC_DIRECT | mitochondrial matrix | 16 | 2.3 | 0.33897712 | 1 | 1 | 0.95620438 |
|  |  |  |  |  |  |  |  |
| **Annotation Cluster 4** | **Enrichment Score: 2.8** |  |  |  |  |  |  |
| **Category** | **Term** | **Cnt.** | **%** | **P-Value** | **Bonferroni** | **Benjamini** | **FDR** |
| UP_KW_BIOLOGICAL_PROCESS | mRNA processing | 38 | 5.4 | 8.47E-08 | 8.64E-06 | 8.64E-06 | 8.13E-06 |
| UP_KW_BIOLOGICAL_PROCESS | mRNA splicing | 29 | 4.1 | 5.49E-06 | 5.60E-04 | 1.87E-04 | 1.76E-04 |
| GOTERM_BP_DIRECT | RNA splicing | 18 | 2.5 | 5.27E-04 | 0.73607555 | 0.29246378 | 0.29234809 |
| GOTERM_BP_DIRECT | mRNA processing | 19 | 2.7 | 5.79E-04 | 0.76884162 | 0.29246378 | 0.29234809 |
| GOTERM_BP_DIRECT | mRNA splicing, via spliceosome | 16 | 2.3 | 0.00229893 | 0.99702761 | 0.64574416 | 0.64548872 |
| UP_KW_CELLULAR_COMPONENT | Spliceosome | 12 | 1.7 | 0.01624919 | 0.48071699 | 0.10832793 | 0.09749514 |
| GOTERM_CC_DIRECT | U2-type precatalytic spliceosome | 6 | 0.8 | 0.02157006 | 0.99999368 | 0.28195145 | 0.26911213 |
| GOTERM_CC_DIRECT | catalytic step 2 spliceosome | 8 | 1.1 | 0.02838101 | 0.99999986 | 0.34624836 | 0.33048113 |
| GOTERM_CC_DIRECT | spliceosomal complex | 10 | 1.4 | 0.03124603 | 0.99999997 | 0.35684373 | 0.34059401 |
| KEGG_PATHWAY | hsa03040:Spliceosome | 14 | 2 | 0.0496819 | 0.99999953 | 0.71710161 | 0.70205752 |
| GOTERM_CC_DIRECT | U2-type catalytic step 2 spliceosome | 4 | 0.6 | 0.07027129 | 1 | 0.55911501 | 0.5336544 |
|  |  |  |  |  |  |  |  |
| **Annotation Cluster 5** | **Enrichment Score: 2.3** |  |  |  |  |  |  |
| **Category** | **Term** | **Cnt.** | **%** | **P-Value** | **Bonferroni** | **Benjamini** | **FDR** |
| GOTERM_CC_DIRECT | cytosolic small ribosomal subunit | 10 | 1.4 | 1.29E-05 | 0.00706815 | 5.91E-04 | 5.64E-04 |
| GOTERM_BP_DIRECT | rRNA processing | 10 | 1.4 | 0.02781694 | 1 | 1 | 1 |
| GOTERM_CC_DIRECT | small-subunit processome | 7 | 1 | 0.03314942 | 0.99999999 | 0.35684373 | 0.34059401 |
| GOTERM_BP_DIRECT | ribosomal small subunit biogenesis | 7 | 1 | 0.03554789 | 1 | 1 | 1 |
|  |  |  |  |  |  |  |  |
| **Annotation Cluster 6** | **Enrichment Score: 2.1** |  |  |  |  |  |  |
| **Category** | **Term** | **Cnt.** | **%** | **P-Value** | **Bonferroni** | **Benjamini** | **FDR** |
| UP_SEQ_FEATURE | DOMAIN:Sm | 7 | 1 | 2.10E-04 | 0.42516899 | 0.13840529 | 0.13840529 |
| INTERPRO | IPR047575:Sm | 7 | 1 | 2.50E-04 | 0.36067628 | 0.44728845 | 0.44728845 |
| GOTERM_BP_DIRECT | spliceosomal complex assembly | 4 | 0.6 | 0.06185992 | 1 | 1 | 1 |
| GOTERM_BP_DIRECT | spliceosomal snRNP assembly | 4 | 0.6 | 0.06739062 | 1 | 1 | 1 |
| GOTERM_CC_DIRECT | SMN-Sm protein complex | 3 | 0.4 | 0.11207614 | 1 | 0.6992023 | 0.66736248 |
|  |  |  |  |  |  |  |  |
| **Annotation Cluster 7** | **Enrichment Score: 2** |  |  |  |  |  |  |
| **Category** | **Term** | **Cnt.** | **%** | **P-Value** | **Bonferroni** | **Benjamini** | **FDR** |
| GOTERM_BP_DIRECT | ER to Golgi vesicle-mediated transport | 13 | 1.8 | 6.94E-04 | 0.82716052 | 0.29246378 | 0.29234809 |
| UP_KW_BIOLOGICAL_PROCESS | ER-Golgi transport | 10 | 1.4 | 0.00985885 | 0.63599688 | 0.16760049 | 0.15774164 |
| GOTERM_CC_DIRECT | transport vesicle | 7 | 1 | 0.09865179 | 1 | 0.68556749 | 0.65434857 |
|  |  |  |  |  |  |  |  |
| **Annotation Cluster 8** | **Enrichment Score: 2** |  |  |  |  |  |  |
| **Category** | **Term** | **Cnt.** | **%** | **P-Value** | **Bonferroni** | **Benjamini** | **FDR** |
| UP_SEQ_FEATURE | DOMAIN:Sm | 7 | 1 | 2.10E-04 | 0.42516899 | 0.13840529 | 0.13840529 |
| INTERPRO | IPR047575:Sm | 7 | 1 | 2.50E-04 | 0.36067628 | 0.44728845 | 0.44728845 |
| GOTERM_CC_DIRECT | precatalytic spliceosome | 6 | 0.8 | 3.47E-04 | 0.17336279 | 0.01269042 | 0.01211254 |
| INTERPRO | IPR010920:LSM_dom_sf | 6 | 0.8 | 7.76E-04 | 0.75094978 | 0.68641661 | 0.68641661 |
| SMART | SM00651:Sm | 5 | 0.7 | 0.00288794 | 0.5090738 | 0.28422657 | 0.28307118 |
| INTERPRO | IPR001163:Sm_dom_euk/arc | 5 | 0.7 | 0.00367886 | 0.99863596 | 1 | 1 |
| GOTERM_CC_DIRECT | Lsm1-7-Pat1 complex | 3 | 0.4 | 0.00962548 | 0.995058 | 0.16513714 | 0.15761724 |
| GOTERM_CC_DIRECT | U12-type spliceosomal complex | 5 | 0.7 | 0.01315935 | 0.99930558 | 0.18783112 | 0.17927779 |
| UP_KW_CELLULAR_COMPONENT | Spliceosome | 12 | 1.7 | 0.01624919 | 0.48071699 | 0.10832793 | 0.09749514 |
| GOTERM_CC_DIRECT | U2-type precatalytic spliceosome | 6 | 0.8 | 0.02157006 | 0.99999368 | 0.28195145 | 0.26911213 |
| GOTERM_CC_DIRECT | catalytic step 2 spliceosome | 8 | 1.1 | 0.02838101 | 0.99999986 | 0.34624836 | 0.33048113 |
| GOTERM_CC_DIRECT | U2-type catalytic step 2 spliceosome | 4 | 0.6 | 0.07027129 | 1 | 0.55911501 | 0.5336544 |
| GOTERM_CC_DIRECT | U4/U6 x U5 tri-snRNP complex | 4 | 0.6 | 0.1745257 | 1 | 0.84067851 | 0.80239625 |
| GOTERM_BP_DIRECT | U2-type prespliceosome assembly | 3 | 0.4 | 0.19552245 | 1 | 1 | 1 |
| GOTERM_CC_DIRECT | U2 snRNP | 3 | 0.4 | 0.22603265 | 1 | 0.95455327 | 0.91108545 |
| GOTERM_CC_DIRECT | U2-type spliceosomal complex | 3 | 0.4 | 0.23798137 | 1 | 0.97501323 | 0.93061372 |
|  |  |  |  |  |  |  |  |
| **Annotation Cluster 9** | **Enrichment Score: 1.9** |  |  |  |  |  |  |
| **Category** | **Term** | **Cnt.** | **%** | **P-Value** | **Bonferroni** | **Benjamini** | **FDR** |
| INTERPRO | IPR008991:Translation_prot_SH3-like_sf | 5 | 0.7 | 0.00153389 | 0.9359297 | 0.68641661 | 0.68641661 |
| SMART | SM00739:KOW | 3 | 0.4 | 0.0179001 | 0.98824307 | 0.62906075 | 0.62650359 |
| UP_SEQ_FEATURE | DOMAIN:KOW | 3 | 0.4 | 0.01907442 | 1 | 1 | 1 |
| INTERPRO | IPR005824:KOW | 3 | 0.4 | 0.04871973 | 1 | 1 | 1 |
|  |  |  |  |  |  |  |  |
| **Annotation Cluster 10** | **Enrichment Score: 1.7** |  |  |  |  |  |  |
| **Category** | **Term** | **Cnt.** | **%** | **P-Value** | **Bonferroni** | **Benjamini** | **FDR** |
| KEGG_PATHWAY | hsa00190:Oxidative phosphorylation | 15 | 2.1 | 3.20E-04 | 0.0873653 | 0.03046833 | 0.02982913 |
| KEGG_PATHWAY | hsa04932:Non-alcoholic fatty liver disease | 13 | 1.8 | 0.01005909 | 0.94450531 | 0.35961258 | 0.35206826 |
| GOTERM_BP_DIRECT | cellular respiration | 6 | 0.8 | 0.01031854 | 1 | 1 | 1 |
| KEGG_PATHWAY | hsa05014:Amyotrophic lateral sclerosis | 23 | 3.2 | 0.01194083 | 0.96779491 | 0.36626192 | 0.3585781 |
| KEGG_PATHWAY | hsa05415:Diabetic cardiomyopathy | 15 | 2.1 | 0.01477716 | 0.98584667 | 0.36626192 | 0.3585781 |
| KEGG_PATHWAY | hsa05012:Parkinson disease | 18 | 2.5 | 0.01553513 | 0.98864298 | 0.36626192 | 0.3585781 |
| KEGG_PATHWAY | hsa04714:Thermogenesis | 16 | 2.3 | 0.02034471 | 0.99720128 | 0.38790589 | 0.37976801 |
| KEGG_PATHWAY | hsa05020:Prion disease | 17 | 2.4 | 0.03661625 | 0.99997674 | 0.61601452 | 0.60309114 |
| KEGG_PATHWAY | hsa05016:Huntington disease | 18 | 2.5 | 0.05014697 | 0.99999959 | 0.71710161 | 0.70205752 |
| UP_KW_CELLULAR_COMPONENT | Mitochondrion inner membrane | 19 | 2.7 | 0.05153749 | 0.87954999 | 0.2290555 | 0.20614995 |
| KEGG_PATHWAY | hsa05010:Alzheimer disease | 21 | 3 | 0.06184149 | 0.99999999 | 0.8102797 | 0.79328082 |
| KEGG_PATHWAY | hsa05022:Pathways of neurodegeneration - multiple diseases | 24 | 3.4 | 0.09101607 | 1 | 1 | 0.97902165 |
| KEGG_PATHWAY | hsa05208:Chemical carcinogenesis - reactive oxygen species | 13 | 1.8 | 0.10738722 | 1 | 1 | 0.98245614 |

1. The Faculty of Medical and Health Sciences , Tel Aviv University, Tel Aviv, Israel [↑](#footnote-ref-2)
2. Department of Physics of Complex Systems, Weizmann Institute of Science, Rehovot 76100, Israel [↑](#footnote-ref-3)
3. Shalvata Mental Health Center, Affiliated with the Faculty of Medicine, Tel-Aviv University, 13 Aliat Hanoar St., Hod Hasharon 45100, Israel

   Correspondence: Libi Hertzberg, The Faculty of Medical and Health Sciences, Tel Aviv University, Tel Aviv, Israel. Email: libi.hertzberg@gmail.com. [↑](#footnote-ref-4)
